# Object Detection and Recognition Based on Deep Learning

**DOI:** 10.3390/s26072200

**Published:** 2026-04-02

**Authors:** Vincenzo Carletti, Alessia Saggese, Paolo Spagnolo

**Affiliations:** 1 Department of Information and Electrical Engineering and Applied Mathematics (DIEM), University of Salerno, 84084 Fisciano, Italy; vcarletti@unisa.it; 2 Institute of Applied Science and Intelligent Systems, National Research Council, 73100 Lecce, Italy; paolo.spagnolo@cnr.it

## 1. Introduction

Object detection and recognition have undergone a profound transformation over the past decade, driven by the rapid evolution of deep learning architectures, the availability of large annotated datasets, and the increasing computational power of modern hardware. From early convolutional neural networks to transformer-based architectures and multimodal perception systems, the field has expanded to address challenges ranging from real-time inference to robustness under occlusion, adverse weather, and domain shift. This Special Issue of *Sensors* brings together contributions that reflect the diversity and maturity of contemporary research in object detection and recognition. The published works span foundational surveys about object detection, with a special focus on Transformer-based detectors, methodological innovations, application-driven solutions, and cross-disciplinary integrations with fields such as structural engineering, fire safety and industrial inspection. Collectively, they illustrate how deep learning continues to push the boundaries of perception, enabling intelligent systems to operate reliably in increasingly complex environments. In the following sections, we provide an integrated overview of the contributions, grouping them into thematic areas that highlight the main research directions represented in this Special Issue.

## 2. Overview of Published Papers

The contributions collected in this Special Issue reflect the remarkable breadth and maturity that deep learning-based object detection and recognition have achieved in recent years. Despite sharing a common methodological foundation, the published works address highly diverse application domains, ranging from fire and smoke detection to structural monitoring, electromagnetic sensing, and FPGA acceleration, demonstrating the versatility and cross-disciplinary impact of modern computer vision approaches.

Notably, four different papers proposed in this Special Issue introduce novel algorithms for flame and smoke detection, a domain where early recognition is essential for safety-critical interventions and in which interest has grown a lot in recent years [1,2,3], which is also due to the release of publicly available datasets and benchmarks on this topic [4]. The authors in the first contribution propose a hybrid visual sensor that combines motion analysis, color filtering, and CNN-based patch classification to improve robustness in outdoor environments where smoke can be easily confused with fog, clouds, or dust. The importance of the temporal information for the detection of flame and smoke has been also explored in the second contribution, where the authors address the specific complexities of wildfire detection in power transmission corridors, introducing an improved Oriented R-CNN, enhanced with metric learning and content-aware upsampling, to better discriminate smoke from visually similar backgrounds. Inspired by the success achieved by attention modules in Transformer-based architectures, Ejaz et al., in the third contribution, further advance fire recognition by integrating channel-wise attention into MobileNetV2, evaluating the impact of data augmentation, color spaces, and adversarial perturbations, and demonstrating improved resilience in challenging visual conditions. An attention-based mechanism has been also used in fourth contribution: the authors introduce YOLO-AR, an enhanced YOLOv8 variant tailored for ancient architecture, where occlusion and dense structural elements complicate fire detection; the authors integrate a Convolutional Block Attention Module (CBAM) at the end of the backbone network and Repulsion Loss with the aim to explicitly optimize bounding box localization accuracy in occluded and dense scenarios.

A second group of papers explores object detection in industrial and manufacturing contexts, where data scarcity, occlusion, and structural complexity pose several challenges. As confirmed by a recent survey on the topic [5,6], it has become, in recent years, a topic of increasing interest for the scientific community. Great interest has been shown towards the problem of defects detections, which has been addressed in two different papers, namely the fifth and the sixth contributions. Qin et al., in the fifth contribution, propose LarGAN, a progressive generative adversarial network capable of synthesizing high-quality defect images from a single sample. Their label auto-rescaling strategy enables effective augmentation of rare defect classes, improving downstream detection performance in steel casting inspection. In the sixth contribution, the authors propose a YOLOv8-based approach applied over a 2D projection of 3D point cloud to enable the automatic detection and segmentation of geometric deviations in industrial steel buildings. YOLOv8 has been also used in Wang and Fan, in the seventh contribution, but with a different aim, namely to extract structural primitives from images of printed plane-frame schematics. The extracted information is then fed into a matrix displacement solver to automatically compute internal forces and generate bending moment diagrams. This work exemplifies how deep learning can automate traditionally manual engineering workflows, opening up new possibilities for intelligent educational tools and rapid structural verification.

In the eighth contribution, Li et al. present a real-time adaptive treadmill system for animal gait analysis, where a lightweight MobileNetV2-based detector, called FOMO, is used to track animals without markers.

Other than the visual domain and point cloud domain, in the ninth contribution, Hosseinzadeh et al. investigate object recognition through electromagnetic backscattering in the millimeter-wave band, employing a 1D CNN to classify objects based on their frequency–domain signatures. Their results highlight the potential of deep learning to complement or replace vision-based recognition in privacy-sensitive or visually degraded environments.

The tenth contribution proposes a resource-efficient YOLOv2 accelerator implemented on a Zynq-7000 FPGA, demonstrating how architectural optimization, quantization, and data-reuse strategies can enable real-time object detection on low-power embedded platforms.

Finally, two surveys have been also proposed within this Special Issue, both focusing on object detection. In the eleventh contribution, the authors analyze the evolution of object detectors, from traditional machine learning approaches (such as Viola-Jones) and two-stages methods of the CNN Era, until one-stage methods (such as YOLO Series and SSD). On the other hand, the authors in the twelfth contribution mainly focus on the breakthrough due to Transformer-based architectures, with End-to-End Object Detection with Transformers (DETR) and its variants, arriving in their discussion to Open-Vocabulary Object Detection tasks.

## 3. Conclusions

The papers gathered in this Special Issue collectively illustrate the rapid evolution and expanding applicability of deep learning-based object detection and recognition. Across diverse domains, ranging from fire safety and industrial inspection to structural engineering and sensing technologies, the contributions demonstrate how modern architectures can be adapted to domain-specific constraints such as occlusion, low data regimes, complex backgrounds, and real-time requirements. Several works advance the state of the art in safety-critical fire and smoke detection, while others introduce innovative solutions for industrial monitoring, including generative augmentation for rare defects and animal monitoring. Additional studies broaden the scope of object detection beyond traditional imaging, leveraging electromagnetic signatures and hardware-efficient FPGA implementations.

Overall, the Special Issue underscores both the methodological maturity and the growing interdisciplinary impact of deep learning-based detection systems, pointing toward future research that unifies sensing, computation, and intelligent automation across increasingly diverse application scenarios.
